# Evaluation of maSSS/maSES-PEG2-RM26 for their potential therapeutic use after labeling with Re-188. Could their [^99m^Tc]Tc-labeled counterparts be used to estimate dosimetry?

**DOI:** 10.1186/s41181-024-00326-3

**Published:** 2025-01-17

**Authors:** Panagiotis Kanellopoulos, Quanyi Yu, Abouzayed Abouzayed, Ekaterina Bezverkhniaia, Vladimir Tolmachev, Anna Orlova

**Affiliations:** 1 Department of Medicinal Chemistry, Uppsala University, Uppsala, 751 23 Sweden; 2 Department of Immunology, Genetics and Pathology, Uppsala University, Uppsala, 751 83 Sweden; 3 Science for Life Laboratory, Uppsala University, Uppsala, 752 37 Sweden

**Keywords:** Bombesin, Tc-99m, Re-188, Antagonist, Radiopharmaceuticals, GRPR, Dosimetry, Estimation, PC-3, Prostate cancer

## Abstract

**Background:**

Gastrin releasing peptide receptor (GRPR)-directed radiopharmaceuticals for targeted radionuclide therapy may be a very promising addition in prostate and breast cancer patient management. Aiming to provide a GRPR-targeting theranostic pair, we have utilized the Tc-99m/Re-188 radiometal pair, in combination with two bombesin based antagonists, maSSS-PEG2-RM26 and maSES-PEG2-RM26. The two main aims of the current study were (i) to elucidate the influence of the radiometal-exchange on the biodistribution profile of the two peptides and (ii) to evaluate the feasibility of using the [^99m^Tc]Tc labeled counterparts for the dosimetry estimation for the [^188^Re]Re-labeled conjugates.

**Results:**

Both peptides were successfully labeled with Re-188 and evaluated both in vitro and in vivo. In GRPR expressing PC-3 cells, both [^188^Re]Re-labeled peptides displayed high cellular uptake (8.5 ± 0.1% and 5 ± 0.3% of added activity, respectively), heavily GRPR-driven, while retaining the radioantagonistic profile with slow internalization rates. Both agents demonstrated high receptor affinity when loaded with ^nat^Re (7.5 nM and 8 nM, respectively). When tested in vivo in GRPR expressing PC-3 xenografts, both radioantagonists demonstrated high tumor accumulation (6.3 ± 0.5%IA/g and 5 ± 1%IA/g at 1 h pi, respectively), with good retention over time (4 ± 2%IA/g and 3.1 ± 0.1%IA/g at 4 h pi, respectively). In addition, their biodistribution profiles were closely mimicking their [^99m^Tc]Tc-labeled counterparts. Statistically significant lower tumor uptake was found for both conjugates labeled with Tc-99m, which may result in underestimation of the dose delivered to the tumor.

**Conclusions:**

All the results indicate that Tc-99 m could be used for dosimetry evaluation for the two [^188^Re]Re-labeled radioligands, with minimal alterations in their biodistribution pattern and tumor targeting capabilities.

**Supplementary Information:**

The online version contains supplementary material available at 10.1186/s41181-024-00326-3.

## Background

For over two decades, nuclear medicine is trying to provide individualized treatment for cancer patients, utilizing alpha- and beta-emitting nuclides. Targeted radionuclide therapy (TRT) aims on delivery of radiotoxic payloads at the sites of the malignancy by employing radioconjugates targeting at biomolecules overexpressed on tumor cells or in their vicinity (Hoefnagel 1991; Taunk et al. 2024). One such molecular target is gastrin releasing peptide receptor (GRPR). GRPR is a member of G-protein coupled receptors (GPCRs) family and is known to be overexpressed in various tumors, such as prostate, breast and gastrointestinal cancers (Baun et al. 2024; D’Onofrio et al. 2023; Dalm et al. 2024; Jensen et al. 2008; Reubi et al. 2004; Reubi and Waser 2003).

GRPR is a promising target for development of radiopharmaceutical, however, quite a few attempts to develop GRPR-targeting radioligands for therapy were made over the years. Initially the focus was on the development of diagnostic agents using of radioagonists, based either of the native gastrin-releasing peptide or its amphibian counterpart, bombesin. This approach was abandoned due to the side effects following the administration of these GRPR agonists (Bodei et al. 2007). To mitigate issues like that, the field shifted towards using GRPR-antagonists as templates for the development of novel radiopharmaceuticals (Baun et al. 2024; Cescato et al. 2008; D’Onofrio et al. 2023; Dalm et al. 2024; Kurth et al. 2020).

**Fig. 1:**
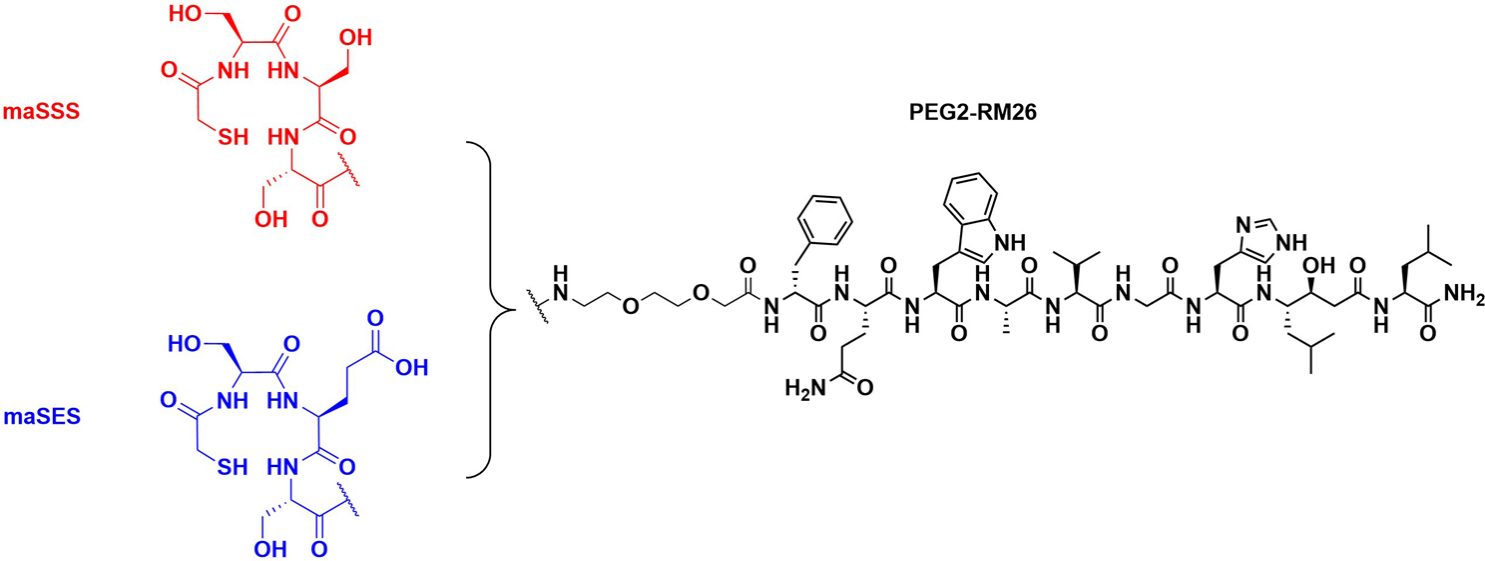
Chemical structures of maSSS-/maSES-PEG2-RM26

One such antagonist is RM26 (D-Phe-Gln-Trp-Ala-Val-Gly-His-Sta-Leu-NH_2_), a 10-amino acid peptide sequence with high affinity for GRPR (Mansi et al. 2009). We recently presented two new analogs utilizing RM26 motif as a receptor targeting sequence, bearing a PEG2 (amine-PEG2-CH_2_COOH) linker and N_3_S amino acid-based chelators for Tc-99m labeling (Abouzayed et al. 2021). The two analogs, maSSS-PEG2-RM26 (mercaptoacetyl-Ser-Ser-Ser-PEG2-RM26) and maSES-PEG2-RM26 (mercaptoacetyl-Ser-Glu-Ser-PEG2-RM26), depicted in Fig. 1, were successfully labeled with Tc-99m and preclinically evaluated (Abouzayed et al. 2021). The promising performance of [^99m^Tc]Tc-maSSS-PEG2-RM26 in PC-3 cells and animal model, led to its clinical testing in prostate and breast cancer patients (Chernov et al. 2023), where it demonstrated no adverse effects and the ability to visualize GRPR-expressing lesions, even previously unknown bone metastasis in a patient.

Making it the next logical step to be the evaluation of the abovementioned analogs labeled with the therapeutic radionuclide Re-188, in an attempt to provide a complete and clinically useful theranostic pair. The chemical similarities of Tc-99m (half-life: 6 h) and Re-188 (half-life: 16.9 h) make them a very appealing isotopes pair, since both could be incorporated to the same N_3_S-chelators and both have clinically relevant logistics with availability through generator-elution (Deutsch et al. 1986; Kleynhans et al. 2023). Both Tc-99m (atomic number: 43, seventh group, fifth period, ionic radius at oxidation state + 5: 74 pm) and Re-188 (atomic number: 75, seventh group, sixth period, ionic radius at oxidation state + 5: 72 pm) are forming complexes with the N_3_S-chelators at oxidation state of + 5, by forming a distorted pyramidal shape around the monooxo-metal core. Despite in chemical and physical similarities of Tc-99m and Re-188 (e.g. possible oxidation states and size, due to the lanthanide contraction), some adjustments on the composition of the labeling conditions are necessary. Specifically, the amount of reducing agent necessary to get monooxo-metal core should be adjusted for transitioning from [^99m^Tc]Tc-labeling to [^188^Re]Re-labeling. Technetium is quite more susceptive to reduction than rhenium, requiring less amount of reducing agent tin chloride to transition from the oxidation state of + 7 to + 5, a key step in its complexation process with the N_3_S-system (Cyr et al. 2007; Gourni et al. 2009; Guhlke et al. 1998; Moura et al. 2006; Tokita et al. 2001). In addition, the two radiometals have different emission profiles making them suitable either for therapy or imaging. For instanse, Tc-99m is emitting mostly γ-rays, with the most abundant being at 140.5 keV (89%), making it ideal for SPECT imaging. On the contrary, Re-188 has mostly beta emissions with electrons average energy at 784 keV and maximum at 2.12 MeV and a low abudance γ-ray emission at 144.05 keV (15.05%), making it better suited for therapeutic purposes (Deutsch et al. 1986; Kleynhans et al. 2023).

The first objective we had to tackle in this study was the establishment of a reproducible radiolabeling protocol for Re-188. Further, the two major questions we aimed to answer in the present work were: (i) does the exchange of the radiometal influence and to what extent the performance of the two analogs? (ii) could the [^99m^Tc]Tc-labeled counterparts be used as valid options for dosimetry estimation for rhenium-186/188 radiotherapy? Thus, after successful labeling with Re-188, both [^188^Re]Re-maSSS-PEG2-RM26 and [^188^Re]Re-maSES-PEG2-RM26 were tested in vitro and in vivo against the imaging agents [^99m^Tc]Tc-maSSS-PEG2-RM26 and [^99m^Tc]Tc-maSES-PEG2-RM26 (Abouzayed et al. 2021) for direct comparison.

## Materials and methods

### Chemicals, reagents and equipment

The peptides maSSS-PEG2-RM26, maSES-PEG2-RM26 and NOTA-PEG2-RM26 were synthesized according to our specs by Pepmic Co., Ltd. (Suzhou, China) and they were kept in aliquots at -20 ^o^C, in MQ water at a 1 mM concentration. PC-3 cells (GRPR-positive prostate cancer cells) were acquired from American Type Culture Collection (ATCC) (Manassas, VA, USA). Roswell Park Memorial Institute (RPMI) 1640 cell growth medium, fetal bovine serum, penicillin-streptomycin, 6-well and 12-well plates were purchased from VWR International (Radnor, PA, USA). Trypsin – 0.25% EDTA solution was from Biochrom AG (Berlin, Germany). [^125^I]I-Tyr4-Bombesin was from PerkinElmer (Waltham, MA, USA). All other reagents were of chemical grade supplied by Merck (Merck Life Science AB, Solna, Sweden). Re-188 was acquired by elution of an OncoBeta^®^^188^W/^188^Re generator (OncoBeta^®^ GmbH, Garching, Germany) in the form of [^188^Re]NaReO_4_. Tc-99m ([^99m^Tc]NaTcO_4_) was acquired by elution of a ^99^Mo/^99m^Tc generator by Mallinckrodt Inc. (ST.Louis, MO, USA).

The HPLC used was equipped with a LaPrep Sigma HPLC LP1100 pump (Hitachi High-Tech Corporation, Hitachinaka, Ibaraki, Japan), a 40D LWL UV-detector with a 4 µL flow cell (Knauer, Berlin, Germany), a Flow scan radioactivity detector (Bioscan) with an FC-3300 NaI/PMT radioactivity probe (Eckert & Ziegler, Berlin, Germany) and a manual simple injector 7725i (Rheodyne) fitted with a 20 µL loop (IDEX Health & Science, LLC, CA, USA). The reverse phase column utilized was a Luna C18 column (5 μm, 100 Å, 150 × 4.6 mm, Phenomenex, Værløse, Denmark). A gradient system was used for elution, starting at 95% H_2_O (0.1% v/v TFA) (A) / 5% acetonitrile (0.1% v/v TFA) (B) and reaching 40% A / 60% B over 20 min.

For measurements of the radioactivity content of all the samples a Wizard2TM gamma counter was used, purchased from PerkinElmer (Hägersten, Sweden).

### Re-188/ Tc-99 m labeling and complex stability studies

For [^188^Re]Re-labeling the reaction mixture consisted of 25 µl of the peptide stock solution, 1.3 mg of SnCl_2_, 65 µl sodium gluconate (1 M), 50 µl of NaHCO_3_ (0.5 M) / Na_2_CO_3_ (0.5 M) 4:1 (pH 9), 14.5 µl Na_2_-EDTA (20 mM). Following the addition of 900 µl of generator eluate (62 ± 5 MBq), the reaction mixture was heated at 90 ^o^C for 1 h. Radiochemical yields and the presence of [^188^Re]ReO_2_ were determined using instant thin layer chromatography (iTLC) with glass microfiber chromatography paper impregnated with silica gel (Agilent Technologies, Santa Clara, CA, USA).

The mobile phases used were (a) phosphate buffered saline (PBS) to determine the amount of free [^188^Re]ReO_4_^−^ (solvent frond, R_f_ = 1; RCY) and (b) a mix of pyridine / acetic acid / water (5:3:1.5) in order to estimate the presence of [^188^Re]ReO_2_ (application point, R_f_ = 0). iTLC stripes were analyzed on Cycle^®^ Plus phosphor imager (PerkinElmer, Hägersten, Sweden). Radiochemical purity after labeling was determined by reverse phase high performance liquid chromatography (system given below).

Similar approach was followed as above for tagging the peptides with ^nat^Re. Instead of the generator eluate, 75 µl of NaReO_4_ 1 mM solution in MQ water was used. The tagging process was monitored by reverse phase HPLC.

The complex stability was tested in 300-fold molar excess of Cysteine (Cys) in PBS. In short, 5 µl of the labeling solution (corresponding to approximately 120 pmol of peptide) were dissolved in 36 µl PBS containing 1 mM of Cys. The mixture was incubated for 1 h at room temperature and the release of Re-188 from the complex was estimated by iTLC.

Labeling with Tc-99 m was performed as previously described (Abouzayed et al. 2021).

### Cell cultures

PC-3 cells were cultured in RPMI medium (glutamine containing) supplemented with 10% w/w fetal bovine serum (FBS) and 1% penicillin (10,000 U/ ml) – streptomycin solution (10,000 µg/ml). Cells were kept at 37 ^o^C, 5% CO_2_ using a Sanyo MCO-19AIC incubator (SANYO Electric Co., Ltd, Osaka City, Osaka, Japan). Trypsin – 0.25% EDTA solution was used for subculturing the cells when they reached approximately 95% confluency.

### In vitro specificity and cellular uptake

For receptor specificity test, 0.7 × 10^6^ cells per well were seeded in 6-well plates. After a wash with PBS, the cells were incubated with 1 mL of solution containing complete growth medium, 0.25 nM of the radiopeptide under evaluation and in the case of “Block” samples, 25 nM of NOTA-PEG2-RM26. After 1 h incubation at 37 ^o^C, the supernatant was discarded and the cells were collected using trypsin – EDTA solution.

To determine cellular uptake of the two radioconjugates, 1 × 10^6^ PC-3 cells/well were seeded a day in advance in 6-well plates and left to proliferate to a uniform monolayer overnight. The following day, growth medium was aspirated and the cells were wash with 1 mL of PBS. A solution containing 1 nM of the radiopeptide under evaluation in complete growth medium was introduced and cells were left to incubate at 37 ^o^C. At pre-determined time points the supernatant was aspirated, the cells were rinsed with 1 ml of cold PBS buffer and incubated glycine buffer (glycine 0.2 M, NaCl 0.15 M, urea 4 M, pH 2, 4 ^o^C) for 5 min and the solution was collected (membrane bound fraction). Following a washing with 1 mL PBS, cells were lysated using NaOH 1 M (internalized fraction).

Radioactivity measurements for the samples was performed using a Wizard2TM gamma counter. For statistical analysis one-way ANOVA with Tuckey’s post hoc analysis was performed using GraphPad Prism v10 for Windows (GraphPad Software, Boston, Massachusetts USA).

### Competition binding experiments

For competition binding experiments live PC-3 cells were used. Using 12-well plates, 5 × 10^5^ PC-3 cells were seeded in each well a day prior to the experiment. The day after, the supernatant was removed followed by washing of the cells with cold PBS (4 ^o^C). Next 350 µL of PBS − 1% w/v bovine serum albumin (BSA) solution was added in the wells followed by 50 µl of the compound under investigation in the same buffer with increased concentrations (0 to 5000 nM). Finally, 100 µl of [^125^I]I-Tyr^4^-BBN (100,000 cpm, 24.6 fmol) in PBS − 1% w/v PBS solution were added and the cells were left to incubate at 4 ^o^C for 5 h. Following a wash with PBS (4 ^o^C), cells were treated with trypsin – EDTA solution and collected. Sample radioactivity was determined with a gamma counter and data analysis and curve fitting were done using GraphPad Prism 7 and a nonlinear regression model.

The statistical significance of the difference between the IC50s for the three conjugates tested was determined by one-way ANOVA with Tuckey’s post hoc analysis using GraphPad Prism.

### Biodistribution studies

Animal studies were in compliance with the European guidelines for laboratory animal protection. BALB/C nu/nu mice were used and the experimental protocols were approved by the ethics committee for animal research in Uppsala (Sweden); permit number 00473/21.

The biodistribution profiles and tumor targeting of maSSS-/SES-PEG2-RM26, labeled with Re-188 or Tc-99 m, were compared head-to-head in the same batch of animals, inoculated with the same PC-3 cells-suspension. After acclimatization, animals were inoculated subcutaneously (right hind leg) with 7 × 10^6^ freshly harvested PC-3 cells per animal, in PBS. After approximately four weeks solid tumors were present at the inoculation sites and the animals were randomly divided in groups of four.

For the ex vivo biodistribution experiments animals were injected via the tail vein with 50 pmol (120 kBq for Re-188 and 60 kBq for Tc-99 m) of the radiopeptide under evaluation, in 100 µl solution in PBS supplemented with 1% w/v BSA. Animals were euthanized at 1 h and 4 h post injection (pi) and tissues / organs of interest and tumors were collected, weighted and their radioactivity content was measured on a gamma counter. Each group comprised by four animals (*n* = 4).

Statistical analysis was performed using GraphPad Prism, employing a two-way ANOVA test with Tuckey’s posthoc analysis.

## Results

### Radiolabeling and complex stability studies

Both peptides were successfully labeled with Re-188, high radiochemical yields and low colloid-formation were confirmed by iTLC (Table 1). The metal-chelate complexes were stable, with less than 2% (determined by iTLC) of Re-188 being released after 1 h incubation in 300x molar excess of cysteine (Cys) (Table 1). Radioconjugates with radiochemical purities (RCP) > 90%, determined both by iTLC and radio-HPLC analysis (Figure S1)) were used for the in vivo experiments.

**Table 1:** Radiochemical characteristics for [^188^Re]Re-maSSS-PEG2-RM26 and [^188^Re]Re-maSES-PEG2-RM26

	[^188^Re]Re-maSSS-PEG2-RM26	[^188^Re]Re-maSES-PEG2-RM26
**RCY % (iTLC)**	89 ± 9	88 ± 9
**Colloids (iTLC)**	0.5 ± 0.1	0.6 ± 0.3
**Re-188 released against Cys (iTLC)**	< 2%	< 2%
**RCP (HPLC)**	> 91%	> 92%
**T**_**R**_ **(HPLC)**	14.9 min	15 min

Radiochemical purities (RCP): the lowest radiochemical purity, as determined by radio-HPLC analysis, for the labeled peptides used for in vivo experiments. Radiochemical yields (RCY) weres determined by iTLC analysis. T_R_: retention time of the radioconjugate’s peak during radio-HPLC analysis

### In vitro specificity and cellular uptake

**Fig. 2:**
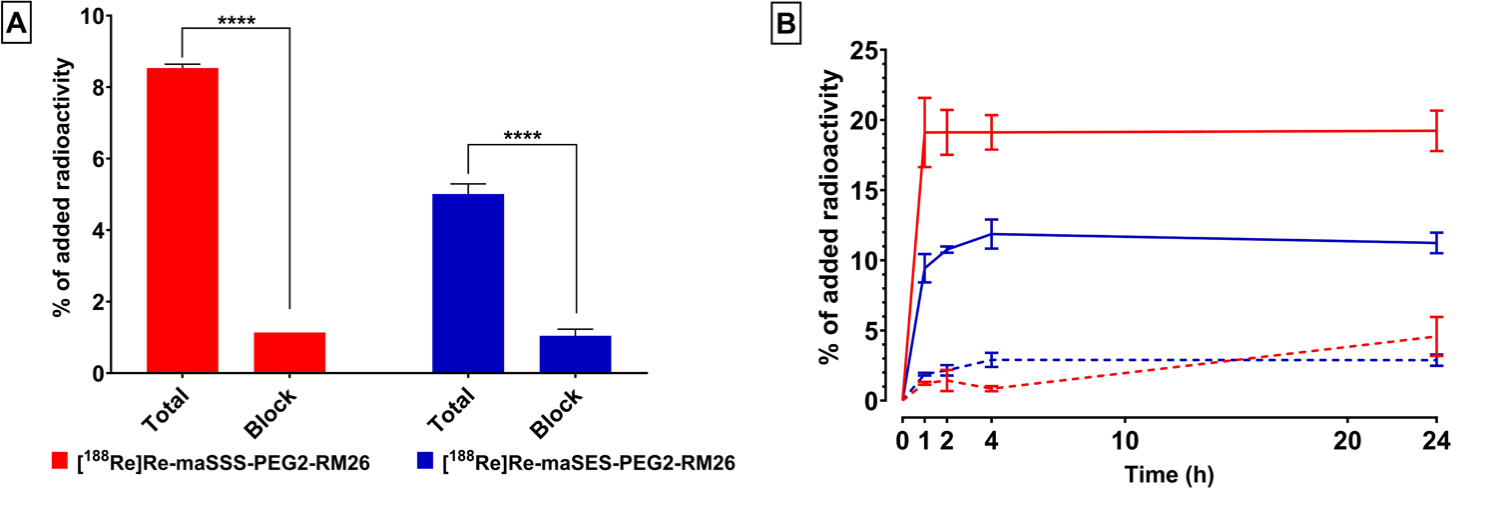
(**A**) In vitro receptor specificity for [^188^Re]Re-maSSS-PEG2-RM26 and [^188^Re]Re-maSES-PEG2-RM26 in PC-3 cells; (**B**) cellular uptake over time for the two radioligands, membrane bound fraction is depicted with the solid lines while the dashed lines represent the internalized fraction

Both radioconjugates displayed highly specific GRPR-binding, as it is demonstrated by the statistically significant decrease of the cell associated activity after blocking of the receptors with excess of the highly-affine NOTA-PEG2-RM26 (*p* < 0.0001 in both cases, Fig. 2A). Between the two analogs [^188^Re]Re-maSSS-PEG2-RM26 displayed higher cell uptake (24 ± 3% of added radioactivity) over the [^188^Re]Re-maSES-PEG2-RM26 (14 ± 0.4% of added radioactivity at 24 h). Over time the majority of the cell associated activity remained bound on the membrane, with a lesser portion being slowly internalized over time (Fig. 2B). Despite the higher overall uptake of [^188^Re]Re-maSSS-PEG2-RM26 (19 ± 4% of cell associated radioactivity), [^188^Re]Re-maSES-PEG2-RM26 had faster internalization, as ratio over the cell-associated radioactivity (21 ± 3% of cell associated radioactivity).

### Competition binding experiments

**Fig. 3:**
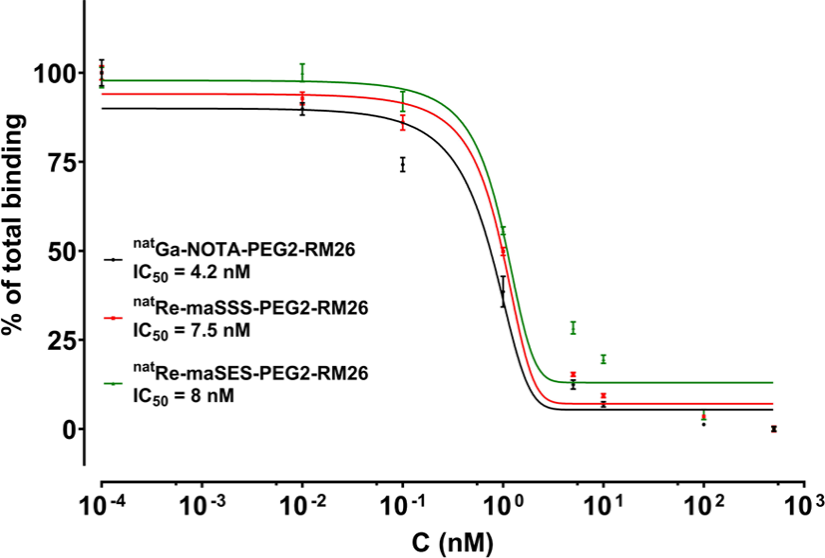
Competition binding curves against [^125^I]I-Tyr^4^-bombesin for^nat^Re-maSSS-PEG2-RM26 (red), ^nat^Re-maSES-PEG2-RM26 (green) and^nat^Ga-NOTA-PEG2-RM26 (black). The solid lines correspond to the equation fitted to describe the competition binding data, from which the IC_50_values were determined

The two ^nat^Re-tagged peptides were tested against [^125^I]I-Tyr^4^-bombesin in competition binding experiments in living cells. Both metalated conjugates displayed similar IC_50_-values in nanomolar range (^nat^Re-maSSS-PEG2-RM26: 7.5 nM; ^nat^Re-maSES-PEG2-RM26: 8 nM), without having any statistical difference between them. In comparison, in the same set of experiments ^nat^Ga-NOTA-PEG2-RM26 (serving as an internal control) had an IC_50_ value of 4.2 nM, verifying their high affinity for GRPR. Representative curves of these experiments are shown in Fig. 3.

### Biodistribution studies

**Fig. 4:**
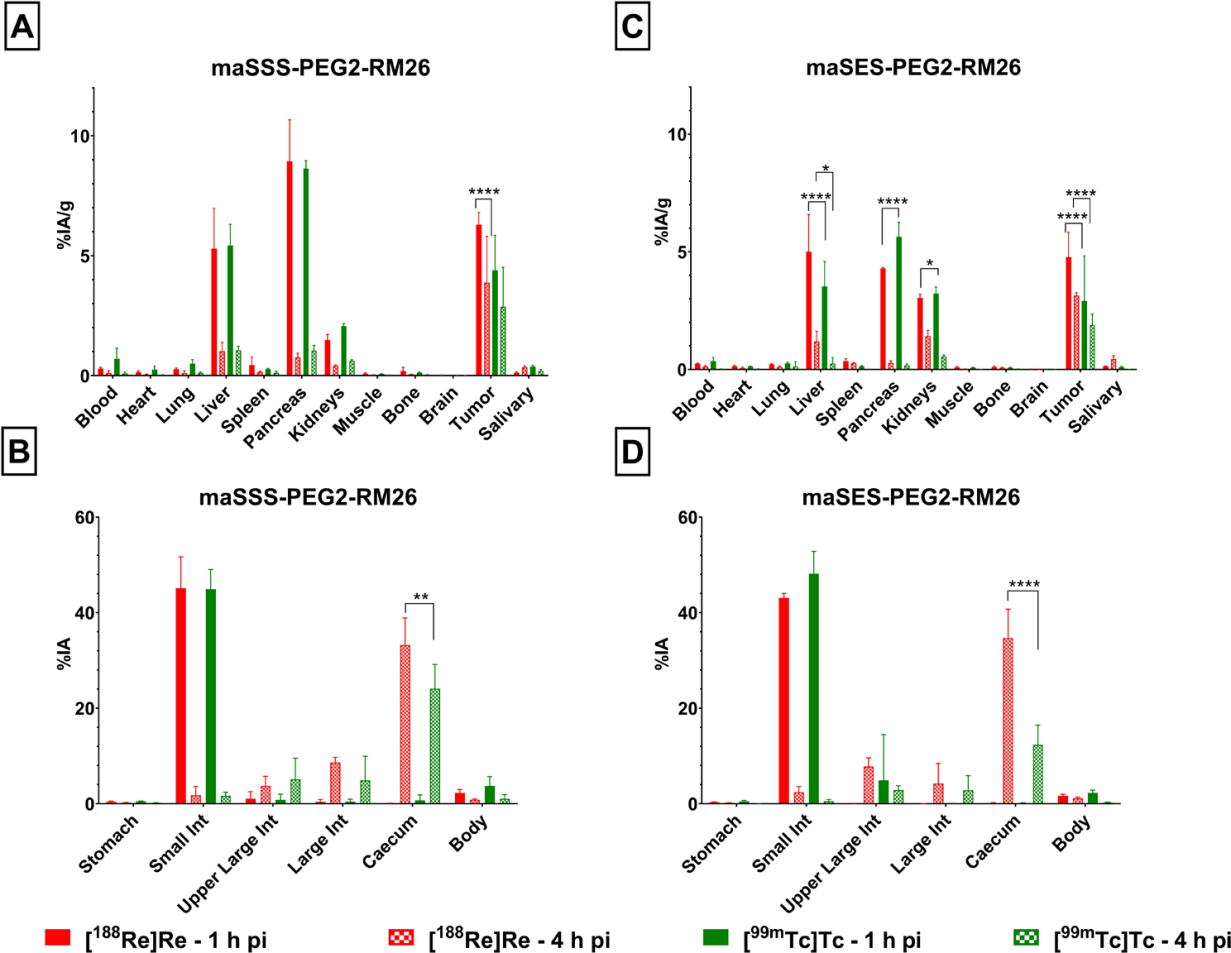
Biodistribution data for (**A** and **B**) maSSS-PEG2-RM26 and (C and D) maSES-PEG2-RM26 labeled with Re-188 (red) and Tc-99 m (green) in PC-3 xenograft bearing mice at 1 h (solid bars) and 4 h (checkered bars) pi. Data in graphs **A** and **C** are given as percentage of injected activity per gram of tissue (%IA/g), while data for the rest of the gastrointestinal track and the carcass (**B**, **D**) are given as percentage of injected activity (%IA). For statistical analysis a two-way ANOVA test was used, with Tuckey’s posthoc analysis. Statistical differences between peptides labeled with Re-188 and Tc-99 m are depicted with stars; *: *p* < 0.05; **: *p* < 0.01; ***: *p* < 0.001 and ****: *p* < 0.0001

The biodistribution profiles of both [^188^Re]Re-maSSS-PEG2-RM26 and [^188^Re]Re-maSES-PEG2-RM26 were compared with their [^99m^Tc]Tc-labeled counterparts at 1 h and 4 h pi. For this set of experiments, the same batch of animals was used to mitigate any biological differences between animals and GRPR expression levels in the xenografts. Biodistribution data are presented in Fig. 4 and summarized in Tables S1 – S2.

The [^188^Re]Re-labeled peptides had the similar uptake-pattern across tissues/organs as their [^99m^Tc]Tc-labeled counterparts. All four compounds had a rapid clearance from blood and the majority of organs/tissues and the carcasses. As can be seen from Figs. 4, [^188^Re]Re-labeled analogs display higher uptake in the xenografts in comparison with their [^99m^Tc]Tc-labeled counterparts. Also, both [^188^Re]Re-maSSS-PEG2-RM26 and [^188^Re]Re-maSES-PEG2-RM26 showed a trend for increased uptake in the salivary glands in later time points, which was not evident for [^99m^Tc]Tc-maSSS-PEG2-RM26 and [^99m^Tc]Tc-maSES-PEG2-RM26.

All four compounds had predominantly hepatobiliary excretion and only for maSES-PEG2-RM26 there was a pronounced difference in the uptake in caecum, between the two different radiometals. At 4 h pi maSES-PEG2-RM26 had 2.3-fold higher values for Re-188 than for Tc-99 m in activity uptake in caecum (35 ± 6%IA vs. 15 ± 6%IA, *p* < 0.0001). In comparison the same ratio for maSSS-PEG2-RM26 was 1.4, which is quite better in terms of prediction.

When [^188^Re]Re-maSES-PEG2-RM26 and [^188^Re]Re-maSSS-PEG2-RM26 were compared, no statistical difference between the compounds at 1–4 h pi could be found for the majority of tissues/organs, with the exception of pancreas (4.29 ± 0.04%IA/g vs. 9 ± 2%IA/g respectively, *p* < 0.001) at 1 h pi.

## Discussion

GRPR has proven a very appealing biomolecular target for imaging and therapy. The introduction of peptide-based GRPR antagonists rekindled the interest for GRPR-targeting radioligands (Baun et al. 2024; D’Onofrio et al. 2023; Dalm et al. 2024). Peptide antagonists like JMV594 (Tokita et al. 2001) and desMet-GRP(6–13) (Wang et al. 1990) gave rise of a new era of GRPR-theranostics, inspiring the development of radioligands such as RM2, NOTA-PEG3-RM26, SB3, DB15 and, of course, NeoBomB1 (Maina et al. 2016; Mansi et al. 2011; MITRAN et al. 2016; Nock et al. 2017, 2021; Varasteh et al. 2014). These developments gave hope for new tools in the clinical management of patients with GRPR-expressing tumors, such as prostate and breast cancer, which have high incidence of GRPR-overexpression (Baun et al. 2024; D’Onofrio et al. 2023; Dalm et al. 2024).

Despite many attempts the most clinically relevant theranostic radionuclide pair, up to this day, is Ga-68 / Lu-177. One promising use of these pair is with NeoBomb1, a bombesin based GRPR-antagonist. Being currently in clinical trials, NeoBomb1 labeled with Ga-68 aims for diagnosis and with Lu-177 for therapy (Duan et al. 2024; Gruber et al. 2020; Nock et al. 2017; Verhoeven et al. 2024). Regardless of the fact that both radiometals can be complexated with DOTA-chelator, their different size and charge density leads to different 3D conformations and compositions of their respective complexes (Price and Orvig 2014). These differences, despite their indifferent appearance, can greatly influence the in vivo behavior of the ligands leading to different receptor-affinities, biodistribution profiles and even stability against peptidases (Lymperis et al. 2018; Maina et al. 2016; Mitran et al. 2019; Varasteh et al. 2015). Plus, the limited availability of PET instrumentation, that limits the use of Ga-68 in specialized hospitals, are hindering the wide use of Ga-68/Lu-177 as a radionuclide pair.

Due to its characteristics and logistics, it is obvious that the Tc-99 m / Re-188 theranostic pair remedies some of the limitations mentioned above. The wide availability of single photon emission computed tomography (SPECT) apparatuses worldwide, the availability of Tc-99 m and Re-188 by their respective generators and the short, but not limiting, half-life of both radionuclides soothes the infrastructure requirements (Knapp et al. 1997; Lamson et al. 1975; Morgat et al. 2017). On top of that, the similar and well-established chemistry of technetium and rhenium, and their similar size and possible oxidations states aid the design of novel radiopharmaceuticals (Cyr et al. 2007; Gourni et al. 2009; Guhlke et al. 1998; Moura et al. 2006; Tokita et al. 2001). The use of MAG3-like chelators, which are suitable for stable binding of Tc-99 m and Re-188 alike, turns the production of single-vial labeling kits into a trivia (Abouzayed et al. 2023).

As a therapeutic radionuclide, Re-188 holds quite appealing attributes. Its availability though a simple elution with saline of a W-188/Re-188 generator makes its clinical use convenient. Its decently long half-life (T_1/2_ = 16,9 h) provides enough time for preparation, administration and in vivo circulation of the radiopharmaceuticals without significant loses of activity outside their tumor-targets. Due to the high energy beta emissions of Re-188, with average energy at 784 keV and maximum at 2.12 MeV, make it more than aqueduct for treatment of malignant tissues. Despite all the above, Re-188 has some shortcoming that should be taken into account. Due to the high energy beta emission of Re-188 (E_βmax_ = 2.12 MeV), the electrons have a tissue penetration range of approximately 11 mm (Lepareur et al. 2019). This fact limits its optimal use for bigger tumors, while for treatment of micro metastases and smaller tumors radionuclides decaying with beta particles with lower energies (e.g. Lu-177 and Tb-161) (Lepareur et al. 2019) or with alpha particles (e.g. Ac-225 or At-211) (Miederer et al. 2024), are preferable. Still, [^188^Re]Re-labeled radiopharmaceuticals can be used in management, aiming in shrinkage of larger lesions, making their subsequent surgical removal possible.

In order to evaluate the feasibility of using the previously reported maSSS/maSES-PEG2-RM26 peptides for therapy, we aimed on testing them, both in vitro and in vivo, after labeling with Re-188. Both peptides were successfully labelled with Re-188, with high radiochemical yields and purities. Both radioligands displayed high metal-chelate stability under cysteine challenge, with very low release of Re-188. This assay is widely used as a surrogate test for prediction of in vivo stability (Hnatowich et al. 1994; Winnard et al. 1996). In GRPR-expressing PC-3 cells, both labeled conjugates were highly GRPR-specific, with [^188^Re]Re-maSSS-PEG2-RM26 having almost double the cell uptake than the maSES-containing analog. When the cell-association over time was evaluated for both [^188^Re]Re-maSSS-PEG2-RM26 and [^188^Re]Re-maSES-PEG2-RM26, a typical profile of a radioantagonist was evident in both cases, with the bulk of the bound radioactivity remaining on the cell membrane and a slow internalization rate over-time. It is worth noting that despite the superior uptake of [^188^Re]Re-maSSS-PEG2-RM26, [^188^Re]Re-maSES-PEG2-RM26 displayed higher internalization rate (19 ± 4% vs. 21 ± 3% of cell associated radioactivity at 24 h, respectively) in PC-3 cells. A possible explanation for the different cellular uptake of the two radioconjugates is their different receptor affinities, while the slightly higher internalization rate of [^188^Re]Re-maSES-PEG2-RM26 may indicate better tumor retention in vivo.

To elucidate the impact of the receptor affinity, both peptides when tagged with ^nat^Re and tested alongside the previously reported ^nat^Ga-NOTA-PEG2-RM26 in competition binding experiments, against [^125^I]I-Tyr^4^-bombesin. Both analogs displayed single-digit nanomolar IC_50_s, with no statistical difference between their affinity for GRPR, while the values were only slightly worse than the ones for ^nat^Ga-NOTA-PEG2-RM26. The higher cellular uptake of [^188^Re]Re-maSSS-PEG2-RM26 in PC-3 cells in combination with the similar affinities of the two analogs, strongly indicate that the exchange of the Ser to Glu in the chelator changes the kinetic profile of the radioligand-receptor interaction while having almost no impact on the affinity. In addition, an order of magnitude faster on-rate constant was reported for [^99m^Tc]Tc-maSSS-PEG2-RM26 than for [^99m^Tc]Tc-maSES-PEG2-RM26 that corroborate with in vitro data in this study (Abouzayed et al. 2021).

The biodistribution profiles and in vivo targeting for the two [^188^Re]Re-coupled peptides and their [^99m^Tc]Tc-labeled counterparts were accessed in a head-to-head comparison, using the same batch of animals. The main aim was to verify any alterations in the pharmacokinetic profile after exchanging Tc-99m to Re-188 and the possibility of using Tc-99m as means to accurately predict the dosimetry of the [^188^Re]Re-labeled maSSS/maSES-PEG2-RM26. As it is evident from Fig. 4 and from the Tables S1 and S2, [^188^Re]Re-maSSS-PEG2-RM26 and [^188^Re]Re-maSES-PEG2-RM26 displayed the same uptake pattern in vivo with [^99m^Tc]Tc-maSSS-PEG2-RM26 and [^99m^Tc]Tc-maSES-PEG2-RM26, respectively, being in accordance with other published reports (Guhlke et al. 1998; Orlova et al. 2010; Pham et al. 2024). From these results it is evident that the use of Tc-99m labeled GRPR targeting agents as a surrogate for dosimetry estimation of Re-188 counterparts is feasible.

All four compounds had very low background values, even at 1 h pi, with the exception of organs taking part in hepatobiliary excretion (e.g. liver and gastrointestinal track). Both new agents demonstrated high metal chelate stability in vivo that was in agreement with data for cysteine challenge. The very low values for salivary glands and stomach (organs where free or released Tc-99 m and Re-186/188 tend to accumulate) imply that there is minimal release of both radiometals from both chelators, confirming their suitability for use in vivo. The relatively high initial activity uptake in pancreas, an organ with high endogenous GRPR expression, was drastically decreased by 4 h pi, a typical attribute for GRPR-radioantagonists.

There was also a tendency for a higher uptake in some tissues (e.g. pancreas, tumor) for the [^188^Re]Re-labeled analogs in comparison with the ones labelled with Tc-99 m, especially for GRPR-expressing tissues, a trend also shown for PSMA and somatostatin analogs (Guhlke et al. 1998; Pham et al. 2024). In the majority of cases the differences were not statistically significant. Exceptions were tumors, where there was a statistical difference in uptake at 4 h for both sets of radioligands, with maSES-PEG2-RM26 retaining the statistical difference also at 24 h pi. For maSES-PEG2-RM26 statistical differences could be found for pancreas, liver and kidneys, between 4 h and 24 h pi. Those difference could lead to underestimation of the dosimetry for liver and at later timepoints for kidneys and underestimation of the absorbed dose by pancreas.

When the hepatic activity uptake was considered, the initial high radioactivity accumulation at 1 h pi, drastically dropped at 4 h pi. On the other hand, the activity accumulation values for the gastrointestinal track as a whole, did not change between 1 h and 4 h pi, ranging between 40 and 50% of injected activity. However, there are no statistical differences in activity uptake between the two radiometals, with the exception for caecum, that might lead to underestimation of the dosimetry for the compounds labeled with Re-188. We also should consider, that the content of the intestines should provide some “shielding” from the emitting electrons, making the differences for caecum and the overall activity uptake less of a problem.

Despite the protection provided by the content of the intestines, the high hepatobiliary excretion strongly indicates that further structural changes are needed in order to increase the hydrophilicity of the new analogs(s). Structural interventions, such as incorporation of charged amino acids or other polar parts either into the linker or the chelator are needed. The aim of the structural changes should be the shift of the excretion pattern towards the renal pathway, leading to faster washout from the excretory organs, lowering the radioligand accumulation in intestines, thus lowering the absorbed dose by the patients.

## Conclusions

We can conclude that based on the biodistribution profile of [^188^Re]Re/[^99m^Tc]Tc-maSSS-PEG2-RM26 and [^188^Re]Re/[^99m^Tc]Tc-maSES-PEG2-RM26, the [^99m^Tc]Tc-labeled counterparts are more than aqueduct to be used for dosimetry estimations for both sets of radioligands. In fact, exchanging of radiometals had little effect on the biodistribution profile of both peptides. Also, from the biodistribution results is evident that the [^99m^Tc]Tc-labeled maSSS-PEG2 could provide better dosimetry predictions for its [^188^Re]Re-labeled counterpart than those for maSES-PEG2-RM26.

## Electronic supplementary material

Below is the link to the electronic supplementary material.

Supplementary Material 1

## Data Availability

All data generated or analyzed during this study are included in this published article and its supplementary information files.

## Abbreviations

Cys: Cysteine
DOTA: 1,4,7,10–tetraazacyclododecane–1,4,7,10–tetraacetic acid
GPCR: G–protein coupled receptor
GRPR: Gastrin realeasing peptide receptor
HPLC: High performance liquid chromatography
iTLC: Instant thin layer chromatography
PET: Possitron emission tomography
pi: Post injection
PSMA: Prostate specific membrane antigen
RCP: Radiochemical purity
SPECT: Single photon emission tomography
T_R_: Retention time
TRT: Targeted radionuclide therapy

## References

[CR2] Abouzayed A, Rinne SS, Sabahnoo H, Sörensen J, Chernov V, Tolmachev V, et al. Preclinical evaluation of 99mTc-Labeled GRPR antagonists maSSS/SES-PEG2-RM26 for imaging of prostate Cancer. Pharmaceutics. 2021;13(2):182. 33573232 10.3390/pharmaceutics13020182 PMC7912279

[CR1] Abouzayed A, Borin J, Lundmark F, Rybina A, Hober S, Zelchan R, et al. The GRPR antagonist [99mTc]Tc-maSSS-PEG2-RM26 towards Phase I Clinical Trial: Kit Preparation, characterization and toxicity. Diagnostics. 2023;13(9):1611. 37175001 10.3390/diagnostics13091611 PMC10178091

[CR3] Baun C, Naghavi-Behzad M, Hildebrandt MG, Gerke O, Thisgaard H. Gastrin-releasing peptide receptor as a theranostic target in breast cancer: a systematic scoping review. Semin Nucl Med. 2024;54(2):256–69. 38342656 10.1053/j.semnuclmed.2024.01.004

[CR4] Bodei L, Ferrari M, Nunn A, Llull J, Martano MC. 177Lu-AMBA bombesin analogue in hormone refractory prostate cancer patients: A phase I escalation study with single-cycle administrations. Eur J Nucl Med Mol Imaging. 2007;34(SupplS2):S221.

[CR5] Cescato R, Maina T, Nock B, Nikolopoulou A, Charalambidis D, Piccand V, et al. Bombesin receptor antagonists may be preferable to agonists for Tumor Targeting. J Nucl Med. 2008;49(2):318–26. 18199616 10.2967/jnumed.107.045054

[CR6] Chernov V, Rybina A, Zelchan R, Medvedeva A, Bragina O, Lushnikova N, et al. Phase I trial of [99mTc]Tc-maSSS-PEG2-RM26, a Bombesin Analogue antagonistic to gastrin-releasing peptide receptors (GRPRs), for SPECT Imaging of GRPR expression in malignant tumors. Cancers (Basel). 2023;15(6):1631. 36980517 10.3390/cancers15061631 PMC10046460

[CR7] Cyr JE, Pearson DA, Wilson DM, Nelson CA, Guaraldi M, Azure MT, et al. Somatostatin receptor-binding peptides suitable for Tumor Radiotherapy with Re-188 or Re-186. Chemistry and initial Biological studies. J Med Chem. 2007;50(6):1354–64. 17315859 10.1021/jm061290i

[CR8] D’Onofrio A, Engelbrecht S, Läppchen T, Rominger A, Gourni E. GRPR-targeting radiotheranostics for breast cancer management. Front Med. 2023;10. 10.3389/fmed.2023.1250799 PMC10657217 38020178

[CR9] Dalm S, Duan H, Iagaru A. Gastrin releasing peptide receptors-targeted PET Diagnostics and Radionuclide Therapy for prostate Cancer Management. PET Clin. 2024;19(3):401–15. 38644111 10.1016/j.cpet.2024.03.004

[CR10] Deutsch E, Libson K, Vanderheyden J-L, Ketring AR, Maxon HR. The chemistry of rhenium and technetium as related to the use of isotopes of these elements in therapeutic and diagnostic nuclear medicine. Int J Radiat Appl Instrum Part B Nucl Med Biol. 1986;13(4):465–77. 10.1016/0883-2897(86)90027-9 3793504

[CR11] Duan H, Song H, Davidzon GA, Moradi F, Liang T, Loening A, et al. Prospective comparison of ^68^ Ga-NeoB and ^68^ Ga-PSMA-R2 PET/MRI in patients with biochemically recurrent prostate Cancer. J Nucl Med. 2024;65(6):897–903. 38664016 10.2967/jnumed.123.267017

[CR12] Gourni E, Bouziotis P, Benaki D, Loudos G, Xanthopoulos S, Paravatou-Petsotas M, et al. Structural Assessment and Biological Evaluation of Two N _3_ S bombesin derivatives. J Med Chem. 2009;52(14):4234–46. 19522464 10.1021/jm900360d

[CR13] Gruber L, Jiménez-Franco LD, Decristoforo C, Uprimny C, Glatting G, Hohenberger P, et al. MITIGATE-NeoBOMB1, a phase I/IIa study to Evaluate Safety, Pharmacokinetics, and preliminary imaging of ^68^ Ga-NeoBOMB1, a gastrin-releasing peptide receptor antagonist, in GIST patients. J Nucl Med. 2020;61(12):1749–55. 32332143 10.2967/jnumed.119.238808

[CR14] Guhlke S, Schaffland A, Zamora PO, Sartor J, Diekmann D, Bender H, et al. 188Re- and 99mTc-MAG3 as prosthetic groups for labeling amines and peptides. Nucl Med Biol. 1998;25(7):621–31. 9804043 10.1016/s0969-8051(98)00025-0

[CR15] Hnatowich DJ, Virzi F, Fogarasi M, Rusckowski M, Winnard P. Can a cysteine challenge assay predict the in vivo behavior of 99mTc-labeled antibodies? Nucl. Med Biol. 1994;21(8):1035–44. 10.1016/0969-8051(94)90175-9 9234361

[CR16] Hoefnagel CA. Radionuclide therapy revisited. Eur J Nucl Med. 1991;18(6):408–31. 1879447 10.1007/BF02258432

[CR17] Jensen RT, Battey JF, Spindel ER, Benya RV. International Union of Pharmacology. LXVIII. Mammalian Bombesin receptors: nomenclature, distribution, Pharmacology, Signaling, and functions in normal and Disease States. Pharmacol Rev. 2008;60(1):1–42. 18055507 10.1124/pr.107.07108 PMC2517428

[CR18] Kleynhans J, Duatti A, Bolzati C. Fundamentals of Rhenium-188 Radiopharmaceutical Chemistry. Molecules. 2023;28(3):1487. 36771153 10.3390/molecules28031487 PMC9921938

[CR19] Knapp FF, Beets AL, Guhlke S, Zamora PO, Bender H, Palmedo H, et al. Availability of rhenium-188 from the alumina-based tungsten-188/rhenium-188 generator for preparation of rhenium-188-labeled radiopharmaceuticals for cancer treatment. Anticancer Res. 1997;17(3B):1783–95. 9179235

[CR20] Kurth J, Krause BJ, Schwarzenböck SM, Bergner C, Hakenberg OW, Heuschkel M. First-in-human dosimetry of gastrin-releasing peptide receptor antagonist [177Lu]Lu-RM2: a radiopharmaceutical for the treatment of metastatic castration-resistant prostate cancer. Eur. J. Nucl. Med. Mol. Imaging [Internet]. 2020;47(1):123–35. Available from: http://link.springer.com/10.1007/s00259-019-04504-3 10.1007/s00259-019-04504-3 31482426

[CR21] Lamson ML, Kirschner AS, Hotte CE, Lipsitz EL, Ice RD. Generator-produced 99m TcO4-: carrier free. J Nucl Med. 1975;16(7):639–41. 1151483

[CR22] Lepareur N, Lacœuille F, Bouvry C, Hindré F, Garcion E, Chérel M et al. Rhenium-188 labeled Radiopharmaceuticals: current clinical applications in Oncology and Promising perspectives. Front Med. 2019;6. 10.3389/fmed.2019.00132 PMC6587137 31259173

[CR23] Lymperis E, Kaloudi A, Sallegger W, Bakker IL, Krenning EP, de Jong M et al. Radiometal-Dependent Biological Profile of the Radiolabeled Gastrin-Releasing Peptide Receptor Antagonist SB3 in Cancer Theranostics: Metabolic and Biodistribution Patterns Defined by Neprilysin. Bioconjug. Chem. [Internet]. 2018;29(5):1774–84. Available from: 10.1021/acs.bioconjchem.8b00225 10.1021/acs.bioconjchem.8b00225 29664606

[CR24] Maina T, Bergsma H, Kulkarni HR, Mueller D, Charalambidis D, Krenning EP et al. Preclinical and first clinical experience with the gastrin-releasing peptide receptor-antagonist [68Ga]SB3 and PET/CT. Eur. J. Nucl. Med. Mol. Imaging [Internet]. 2016;43(5):964–73. Available from: https://link.springer.com/10.1007/s00259-015-3232-1 10.1007/s00259-015-3232-1 26631238

[CR25] Mansi R, Wang X, Forrer F, Kneifel S, Tamma M-L, Waser B, et al. Evaluation of a 1,4,7,10-Tetraazacyclododecane-1,4,7,10-Tetraacetic acid–conjugated bombesin-based Radioantagonist for the labeling with single-Photon Emission Computed Tomography, Positron Emission Tomography, and therapeutic radionuclides. Clin Cancer Res. 2009;15(16):5240–9. 19671861 10.1158/1078-0432.CCR-08-3145

[CR26] Mansi R, Wang X, Forrer F, Waser B, Cescato R, Graham K et al. Development of a potent DOTA-conjugated bombesin antagonist for targeting GRPr-positive tumours. Eur. J. Nucl. Med. Mol. Imaging [Internet]. 2011;38(1):97–107. Available from: http://link.springer.com/10.1007/s00259-010-1596-9 10.1007/s00259-010-1596-9 20717822

[CR27] Miederer M, Benešová-Schäfer M, Mamat C, Kästner D, Pretze M, Michler E, et al. Alpha-emitting radionuclides: current status and future perspectives. Pharmaceuticals. 2024;17(1):76. 38256909 10.3390/ph17010076 PMC10821197

[CR29] MITRAN B, VARASTEH Z, SELVARAJU RK, SÖRENSEN LINDEBERGG, LARHED J et al. M,. Selection of optimal chelator improves the contrast of GRPR imaging using bombesin analogue RM26. Int. J. Oncol. [Internet]. 2016;48(5):2124–34. Available from: https://www.spandidos-publications.com/10.3892/ijo.2016.3429 10.3892/ijo.2016.3429 26983776

[CR28] Mitran B, Rinne SS, Konijnenberg MW, Maina T, Nock BA, Altai M et al. Trastuzumab cotreatment improves survival of mice with PC-3 prostate cancer xenografts treated with the GRPR antagonist 177 Lu‐DOTAGA‐PEG 2 ‐RM26. Int. J. Cancer [Internet]. 2019;145(12):3347–58. Available from: https://onlinelibrary.wiley.com/doi/10.1002/ijc.32401 10.1002/ijc.32401 PMC6852655 31077356

[CR30] Morgat C, MacGrogan G, Brouste V, Vélasco V, Sévenet N, Bonnefoi H et al. Expression of Gastrin-Releasing Peptide Receptor in Breast Cancer and Its Association with Pathologic, Biologic, and Clinical Parameters: A Study of 1,432 Primary Tumors. J. Nucl. Med. [Internet]. 2017;58(9):1401–7. Available from: 10.2967/jnumed.116.188011 10.2967/jnumed.116.188011 28280221

[CR31] Moura C, Vítor RF, Maria L, Paulo A, Santos IC, Santos I. Rhenium(v) oxocomplexes with novel pyrazolyl-based N_4_- and N_3_S-donor chelators. Dalt Trans 2006;47:5630–40. Available from: https://doi.org/10.1039/B611034G 10.1039/b611034g 17225899

[CR33] Nock BA, Kaloudi A, Lymperis E, Giarika A, Kulkarni HR, Klette I, et al. Theranostic perspectives in prostate Cancer with the gastrin-releasing peptide receptor antagonist NeoBOMB1: preclinical and first clinical results. J Nucl Med. 2017;58(1):75–80. 27493272 10.2967/jnumed.116.178889

[CR32] Nock BA, Kaloudi A, Kanellopoulos P, Janota B, Bromińska B, Iżycki D et al. [99mTc]Tc-DB15 in GRPR-Targeted Tumor Imaging with SPECT: From Preclinical Evaluation to the First Clinical Outcomes. Cancers (Basel). [Internet]. 2021;13(20):5093. Available from: https://www.mdpi.com/2072-6694/13/20/5093 10.3390/cancers13205093 PMC8533986 34680243

[CR34] Orlova A, Tran TA, Ekblad T, Karlström AE, Tolmachev V. 186Re-maSGS-ZHER2:342, a potential Affibody conjugate for systemic therapy of HER2-expressing tumours. Eur J Nucl Med Mol Imaging. 2010;37(2):260–9. 19771426 10.1007/s00259-009-1268-9

[CR35] Pham TT, Hungnes IN, Rivas C, Cleaver J, Firth G, Blower PJ, et al. Receptor-targeted peptide conjugates based on Diphosphines Enable Preparation of ^99m^ tc and ^188^ re theranostic agents for prostate Cancer. J Nucl Med. 2024;65(7):1087–94. 38844360 10.2967/jnumed.124.267450 PMC11218721

[CR36] Price EW, Orvig C. Matching chelators to radiometals for radiopharmaceuticals. Chem. Soc. Rev. [Internet]. 2014;43(1):260–90. Available from: http://xlink.rsc.org/?DOI=C3CS60304K 10.1039/c3cs60304k 24173525

[CR38] Reubi JC, Waser B. Concomitant expression of several peptide receptors in neuroendocrine tumours: molecular basis for in vivo multireceptor tumour targeting. Eur J Nucl Med Mol Imaging. 2003;30(5):781–93. 12707737 10.1007/s00259-003-1184-3

[CR37] Reubi JC, Körner M, Waser B, Mazzucchelli L, Guillou L. High expression of peptide receptors as a novel target in gastrointestinal stromal tumours. Eur J Nucl Med Mol Imaging. 2004;31(6):803–10. 14985869 10.1007/s00259-004-1476-2

[CR39] Taunk NK, Escorcia FE, Lewis JS, Bodei L. Radiopharmaceuticals for Cancer diagnosis and therapy. Cancer J. 2024;30(3):218–23. 38753757 10.1097/PPO.0000000000000720 PMC11232930

[CR40] Tokita K, Katsuno T, Hocart SJ, Coy DH, Llinares M, Martinez J, et al. Molecular basis for selectivity of High Affinity peptide antagonists for the gastrin-releasing peptide receptor. J Biol Chem. 2001;276(39):36652–63. 11463790 10.1074/jbc.M104566200

[CR42] Varasteh Z, Rosenström U, Velikyan I, Mitran B, Altai M, Honarvar H, et al. The effect of Mini-PEG-Based spacer length on binding and Pharmacokinetic properties of a 68Ga-Labeled NOTA-Conjugated antagonistic Analog of Bombesin. Molecules. 2014;19(7):10455–72. 25036155 10.3390/molecules190710455 PMC6270800

[CR41] Varasteh Z, Mitran B, Rosenström U, Velikyan I, Rosestedt M, Lindeberg G et al. The effect of macrocyclic chelators on the targeting properties of the 68 Ga-labeled gastrin releasing peptide receptor antagonist PEG 2 -RM26. Nucl. Med. Biol. [Internet]. 2015;42(5):446–54. Available from: https://linkinghub.elsevier.com/retrieve/pii/S0969805114005745 10.1016/j.nucmedbio.2014.12.009 25684649

[CR43] Verhoeven M, Haeck J, de Blois E, Orlandi F, Barbato D, Tedesco M, et al. The Balance between the therapeutic efficacy and safety of [177Lu]Lu-NeoB in a preclinical prostate Cancer Model. Mol Imaging Biol. 2024;26(1):114–23. 37640941 10.1007/s11307-023-01851-4 PMC10828073

[CR44] Wang LH, Coy DH, Taylor JE, Jiang NY, Moreau JP, Huang SC et al. des-Met carboxyl-terminally modified analogues of bombesin function as potent bombesin receptor antagonists, partial agonists, or agonists. J. Biol. Chem. [Internet]. 1990;265(26):15695–703. Available from: https://linkinghub.elsevier.com/retrieve/pii/S0021925818554545 1697594

[CR45] Winnard P, Virzi E, Fogarasi M, Rusckowski M, Hnatowich DJ. Investigations of directly labeling antibodies with rhenium-188. Q J Nucl Med. 1996;40(2):151–60. 8909100

