# Supplementary material for: Evaluation of maSSS/maSES-PEG2-RM26 for their potential therapeutic use after labeling with Re-188. Could their [99mTc]Tc-labeled counterparts be used to estimate dosimetry?
Source: EJNMMI Radiopharm Chem. 2025 Jan 17;10:3. doi: 10.1186/s41181-024-00326-3 (PMC11748620; doi:10.1186/s41181-024-00326-3)
Supplement: Supplementary file 1 — Supplementary Material 1 [file 41181_2024_326_MOESM1_ESM.docx]

# Supplementary

# Title

Evaluation of maSSS/maSES-PEG2-RM26 for their potential therapeutic use after labeling with Re-188. Could their [^99m^Tc]Tc-labeled counterparts be used to estimate dosimetry?

# Authors

Panagiotis Kanellopoulos^1*^, Quanyi Yu^1^, Abouzayed Abouzayed^1^, Ekaterina Bezverkhniaia^1^, Vladimir Tolmachev^2^, Anna Orlova^1,3^

1. Department of Medicinal Chemistry, Uppsala University, Uppsala 751 23, Sweden
2. Department of Immunology, Genetics and Pathology, Uppsala University, Uppsala 751 83, Sweden.
3. Science for Life Laboratory, Uppsala University, Uppsala 752 37, Sweden

* Corresponding author

***Table S1****. Biodistribution data for [^188^Re]Re-maSSS-PEG2-RM26 and [^188^Re]Re-maSES-PEG2-RM26 at 1 h and 4 h pi in mice bearing PC-3 xenografts.*

| **Re-188 labelled** | **maSSS-PEG2-RM26** | | **maSES-PEG2-RM26** | |
| --- | --- | --- | --- | --- |
| **%IA/g** | **1 h pi** | **4 h pi** | **1 h pi** | **4 h pi** |
| **Blood** | 0.29 ± 0.04 | 0.10 ± 0.09 | 0.25 ± 0.02 | 0.12 ± 0.04 |
| **Heart** | 0.15 ± 0.03 | 0.04 ± 0.03 | 0.13 ± 0.04 | 0.06 ± 0.03 |
| **Lungs** | 0.27 ± 0.02 | 0.11 ± 0.09 | 0.22 ± 0.03 | 0.11 ± 0.04 |
| **Liver** | 5.3 ± 0.2*^a^* | 1 ± 0.4*^a^* | 5 ± 2*^b^* | 1.2 ± 0.4*^b^* |
| **Spleen** | 0.4 ± 0.4 | 0.14 ± 0.03 | 0.36 ± 0.09 | 0.27 ± 0.02 |
| **Pancreas** | 9 ± 2*^a,c^* | 0.8 ± 0.2*^a^* | 4.29 ± 0.04*^b,c^* | 0.3 ± 0.1*^b^* |
| **Kidneys** | 1.5 ± 0.2*^a^* | 0.41 ± 0.03*^a^* | 3.1 ± 0.2*^b^* | 1.4 ± 0.3*^b^* |
| **Muscle** | 0.08 ± 0.05 | 0.03 ± 0.01 | 0.08 ± 0.04 | 0.02 ± 0.01 |
| **Bone** | 0.2 ± 0.2 | 0.05 ± 0.01 | 0.10 ± 0.03 | 0.08 ± 0.01 |
| **Brain** | 0.01 ± 0.01 | 0.01 ± 0.01 | 0.01 ± 0.01 | 0.01 ± 0.01 |
| **Tumor** | 6.3 ± 0.5*^a^* | 4 ± 2*^a^* | 5 ± 1*^b^* | 3.1 ± 0.1*^b^* |
| **Salivary** | 0.12 ± 0.04 | 0.35 ± 0.06 | 0.13 ± 0.02 | 0.4 ± 0.2 |
| **%IA** |  |  |  |  |
| **Stomach** | 0.4 ± 0.2 | 0.23 ± 0.04 | 0.30 ± 0.09 | 0.17 ± 0.04 |
| **Small Int** | 45 ± 7*^a^* | 2 ± 2*^a^* | 43 ± 0.9*^b^* | 2 ± 1.2*^b^* |
| **Upper Large Int** | 0.3 ± 0.2*^a^* | 4 ± 2*^a,c^* | 0.07 ± 0.02*^b^* | 8 ± 2*^b,c^* |
| **Large Int** | 0.4 ± 0.5 | 9 ± 1*^c^* | 0.05 ± 0.02 | 6 ± 4*^c^* |
| **Caecum** | 0.13 ± 0.01*^a^* | 33 ± 6*^a^* | 0.2 ± 0.1*^b^* | 35 ± 6*^b^* |
| **Body** | 2.3 ± 0.8 | 0.8 ± 0.2 | 1.7 ± 0.4 | 1.1 ± 0.3 |

*a: statistical difference between 1 h and 4 h pi for maSSS-PEG2-RM26; b: statistical difference between 1 h and 4 h pi for maSES-PEG2-RM26; c: statistical difference between maSSS-PEG2-RM26 and maSES-PEG2-RM26 at 4 h pi. Statistical analysis was performed using a two-way ANOVA test with Tuckey’s posthoc analysis.*

***Table S2****. Biodistribution data for* *[^99m^Tc]Tc-maSSS-PEG2-RM26 and [^99m^Tc]Tc -maSES-PEG2-RM26 at 1 h and 4 h pi in mice bearing PC-3 xenografts.*

| **Tc-99m labelled** | **maSSS-PEG2-RM26** | | **maSES-PEG2-RM26** | |
| --- | --- | --- | --- | --- |
| **%IA/g** | **1 h pi** | **4 h pi** | **1 h pi** | **4 h pi** |
| **Blood** | 0.7 ± 0.5 | 0.09 ± 0.06 | 0.36 ± 0.15 | 0.02 ± 0.01 |
| **Heart** | 0.25 ± 0.14 | 0.03 ± 0.01 | 0.12 ± 0.02 | 0.02 ± 0.01 |
| **Lungs** | 0.50 ± 0.17 | 0.11 ± 0.04 | 0.26 ± 0.04 | 0.02 ± 0.01 |
| **Liver** | 5.4 ± 0.9*^a^* | 1.1 ± 0.2*^a^* | 4 ± 1*^b^* | 0.25 ± 0.25*^b^* |
| **Spleen** | 0.27 ± 0.03 | 0.12 ± 0.06 | 0.13 ± 0.03 | 0.01 ± 0.01 |
| **Pancreas** | 8.63 ± 0.32*^a^* | 1.0 ± 0.2*^a^* | 5.64 ±0.61*^b^* | 0.17 ± 0.07*^b^* |
| **Kidneys** | 2.1 ± 0.1*^a^* | 0.62 ± 0.05*^a^* | 3.2 ± 0.3*^b^* | 0.56 ± 0.06*^b^* |
| **Muscle** | 0.07 ± 0.01 | 0.02 ± 0.01 | 0.07 ± 0.02 | 0.01 ± 0.01 |
| **Bone** | 0.12 ± 0.02 | 0.03 ± 0.01 | 0.08 ± 0.01 | 0.02 ± 0.01 |
| **Brain** | 0.01 ± 0.01 | 0.01 ± 0.01 | 0.01 ± 0.01 | 0.01 ± 0.01 |
| **Tumor** | 4.4 ± 1.5*^a^* | 3 ± 2*^a^* | 3 ± 2*^b^* | 1.5 ± 0.8*^b^* |
| **Salivary** | 0.37 ± 0.06 | 0.19 ± 0.07 | 0.10 ± 0.02 | 0.01 ± 0.01 |
| **%IA** |  |  |  |  |
| **Stomach** | 0.47 ± 0.09 | 0.17 ± 0.04 | 0.45 ± 0.22 | 0.03 ± 0.02 |
| **Small Int** | 45 ± 4*^a^* | 1.9 ± 0.5*^a^* | 48 ± 5*^b^* | 0.5 ± 0.4*^b^* |
| **Upper Large Int** | 0.21 ± 0.07*^c^* | 7 ± 4 | 0.11 ± 0.01*^c^* | 2.9 ± 0.9 |
| **Large Int** | 0.5 ± 0.7 | 7 ± 5 | 0.07 ± 0.05 | 3 ± 3 |
| **Caecum** | 0.11 ± 0.02*^a^* | 24 ± 5*^a,d^* | 0.13 ± 0.08*^b^* | 15 ± 6*^b,d^* |
| **Body** | 4 ± 2 | 1.1 ± 0.9 | 2.2 ± 0.6 | 0.25 ± 0.08 |

*a: statistical difference between 1 h and 4 h pi for maSSS-PEG2-RM26; b: statistical difference between 1 h and 4 h pi for maSES-PEG2-RM26; c: statistical difference between maSSS-PEG2-RM26 and maSES-PEG2-RM26 at 1 h pi; d: statistical difference between maSSS-PEG2-RM26 and maSES-PEG2-RM26 at 4 h pi. Statistical analysis was performed using a two-way ANOVA test with Tuckey’s posthoc analysis.*


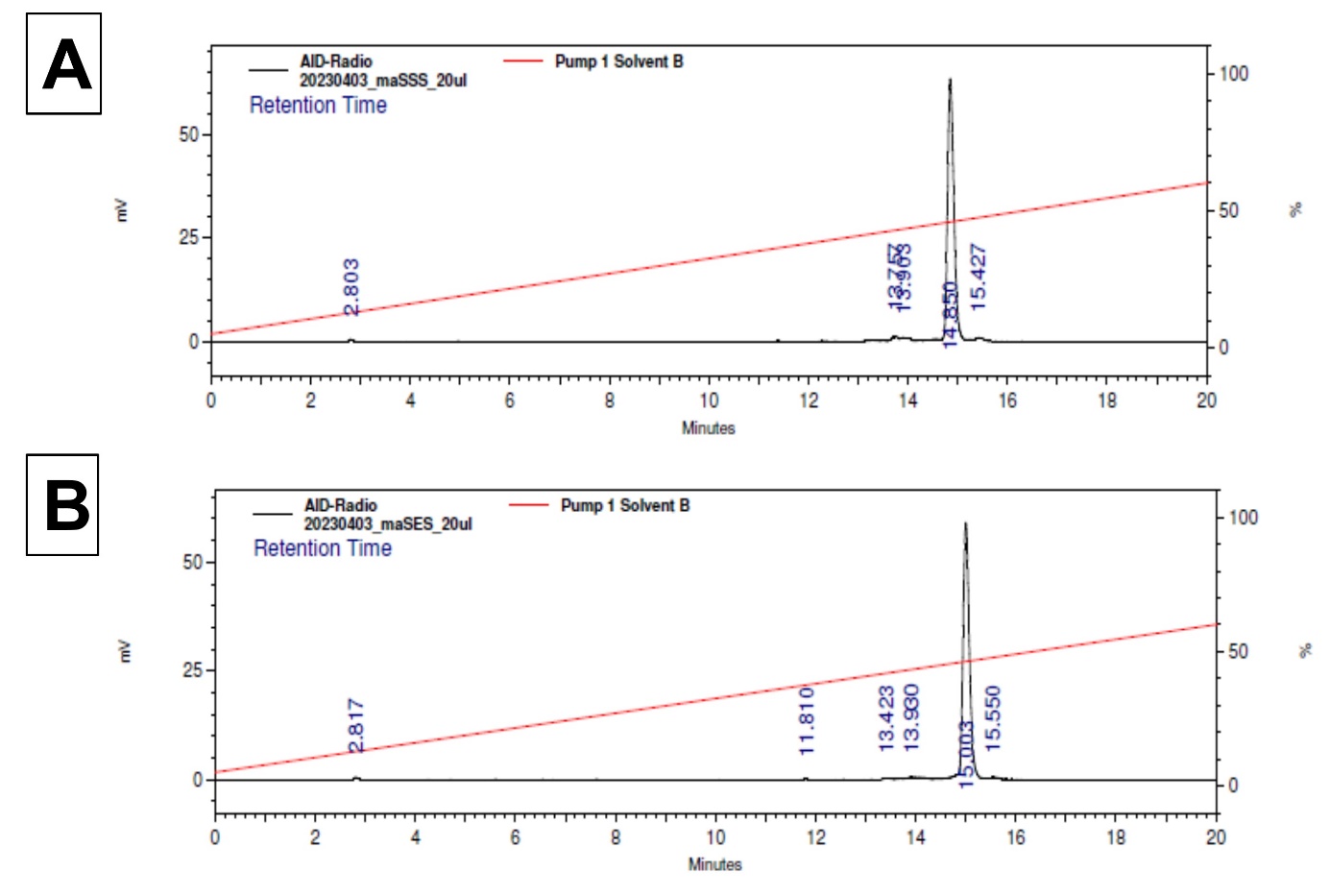


***Figure S1****. Radiochromatograms of (A) [^188^Re]Re-maSSS-PEG2-RM26 and (B) [^188^Re]Re-maSES-PEG2-RM26.*
